# Transportation as a Barrier to Health Care Access Among Patients With Knee Arthritis: Cross-Sectional Study

**DOI:** 10.2196/91207

**Published:** 2026-03-06

**Authors:** Erin Youn, James Chen

**Affiliations:** 1 Advanced Orthopedics and Sports Medicine, 450 Sutter Street Suite 400, San Francisco, CA, 94108, United States, 1 2136050229

**Keywords:** knee, arthritis, transportation delays, access to care, structural barriers

## Abstract

This study uses the 2011-2017 National Health Interview Survey (NHIS) data to demonstrate that sociodemographic factors are associated with transportation delays among individuals with knee osteoarthritis.

## Introduction

In the United States, knee pain, often secondary to various forms of arthritis, is responsible for 5% of all primary care visits [1]. In accessing health care, transportation has often been cited as an impediment to proper care across various diseases and health conditions [2]. While there are studies that document decreased access to health care with respect to transportation barriers [2,3], to the best of our knowledge, there are none that explore this relationship among those diagnosed with knee arthritis.

## Methods

### Study Design

This retrospective study was conducted using data from the 2011‐2017 National Health Interview Survey (NHIS). The NHIS is administered in-person annually and is a cross-sectional survey of noninstitutionalized adults in the United States [4]. Knee arthritis was defined by answers to questions pertaining to self-reported physician-diagnosed knee arthritis as well as self-reported knee pain. Among respondents who were told “by a doctor or other health professional that [they had] some form of arthritis, rheumatoid arthritis, gout, lupus, or fibromyalgia,” knee arthritis was defined by those who indicated that they experienced “symptoms of pain, aching, or stiffness in or around a joint.” (Multimedia Appendix 1). Adults who agreed to the statement, “Have you delayed getting care for any of the following reasons…In the past 12 months…You didn’t have transportation” met the criteria for experiencing barriers to health care due to transportation delays (Multimedia Appendix 1).

Sociodemographic variables were used as covariates. Univariable and multivariable logistic regressions were conducted using R Studio (version 1.4.1106; R Foundation for Statistical Computing), α=0.05. Weighted analysis generated nationally representative results. Participants were excluded from this study if questions regarding knee arthritis or transportation delays were unanswered or if key sociodemographic factors were missing.

### Ethical Considerations

Per National Institute of Health (NIH) guidelines, this research does not meet the definition of human subjects research as this data is publicly available, anonymized, and not obtained for the purposes of this study; therefore, institutional review board approval was not required for this secondary analysis [5].

## Results

A total of 72,930,571 weighted respondents answered the question for knee arthritis on the 2011‐2017 survey (mean age, 53.8, SE 0.10) years. Of the weighted respondents, 62.1% (n=45,258,537; mean age 53.9, SE: 0.12) years indicated that they experienced knee arthritis. Within the knee arthritis cohort, 3.8% (n=1,723,968) of participants noted transportation delays in medical care. Univariable and multivariable logistic regression analyses were performed to assess the effect of various sociodemographic variables on transportation delays for individuals with knee arthritis (Table 1).

**Table 1.** Univariable and multivariable regression analysis of sociodemographic factors and transportation delays among those with knee arthritis.

Variables	Unadjusted odds ratio (95% CI)	Adjusted odds ratio (95% CI)
Sex		
Male	1.00 (Reference)	1.00 (Reference)
Female	*1.69 (1.49‐1.92*)^a^	*1.47 (1.29‐1.67*)^a^
Education		
less than High school	1.00 (Reference)	1.00 (Reference)
High school or GED^b^	*0.49 (0.42‐0.57*)^a^	*0.84 (0.71‐0.99*)^a^
College or more	*0.33 (0.29‐0.37*)^a^	0.85 (0.73‐1.00)
Region		
Midwest	1.00 (Reference)	1.00 (Reference)
Northeast	0.90 (0.72‐1.12)	0.90 (0.72‐1.12)
South	1.23 (1.03‐1.46)	0.97 (0.81‐1.15)
West	1.13 (0.92‐1.38)	1.19 (0.96‐1.46)
Age (y)		
17‐44	1.00 (Reference)	1.00 (Reference)
45‐64	0.98 (0.86‐1.12)	*0.82 (0.70‐0.95*)^a^
>65	*0.73 (0.62‐0.85*)^a^	*0.59 (0.49‐0.72*)^a^
Race		
White	1.00 (Reference)	1.00 (Reference)
Hispanic	*1.62 (1.34‐1.96*)^a^	0.86 (0.70‐1.07)
Black	*2.28 (1.97‐2.64*)^a^	*1.21 (1.04‐1.42*)^a^
Asian	*0.65 (0.45‐0.94*)^a^	*0.50 (0.33‐0.76*)^a^
Other	*2.84 (2.20‐3.67*)^a^	*1.59 (1.22‐2.09*)^a^
Income		
>200%	1.00 (Reference)	1.00 (Reference)
100%‐200%	*4.63 (3.90‐5.50*)^a^	*2.47 (2.02‐3.02*)^a^
<100%	*10.31 (8.77‐12.11*)^a^	*3.81 (3.07‐4.74*)^a^
Insurance		
Private	1.00 (Reference)	1.00 (Reference)
Public	*9.75 (8.26‐11.51*)^a^	*2.87 (2.31‐3.57*)^a^
No insurance	*4.35 (3.50‐5.41*)^a^	*1.83 (1.44‐2.32*)^a^
Other	*3.88 (3.25‐4.64*)^a^	*2.39 (1.95‐2.94*)^a^
Health status		
Bad health	1.00 (Reference)	1.00 (Reference)
Good health	*0.19 (0.17‐0.22*)^a^	*0.37 (0.32‐0.42*)^a^

a Italicized aOR values denote that results that are statistically significant.

b GED: General Educational Development.

Multivariable logistic regression analysis found that Black individuals (adjusted odds ratio [aOR] 1.21; 95% CI 1.04‐1.42), individuals who were 100%‐200% above the federal poverty level (aOR, 2.47; 95% CI 2.02‐3.02), and those<100% of the federal poverty level (aOR, 3.81; 95% CI 3.07‐4.74) were associated with greater odds of encountering transportation delays. Those who were on public insurance (aOR, 2.87; 95% CI 2.31‐3.57), those with no insurance (aOR, 1.83; 95% CI 1.44‐2.32), and those with other insurance (aOR, 2.39; 95% CI 1.95‐2.94) were also more likely to experience transportation delays.

## Discussion

### Principal Findings

Black individuals were associated with increased odds of transportation delays. Studies highlight that residential segregation along racial lines impact Black individuals’ ability to access health care [6]. A retrospective 20-year period study has found that among Black individuals in the United States, a high level of residential segregation coupled with a high density of low-income residents increased the odds of urban hospital closures [7]. Thus, it is plausible that hospital closures and other such factors associated with residential segregation may place these communities further away from medical facilities, which could further exacerbate access to reliable and efficient transportation.

Our study revealed that individuals with an annual income of 100%‐200% above the federal poverty level as well as individuals<100% the federal poverty level experience increased odds of transportation delays. This may reflect difficulties with car ownership among lower-income individuals, as previous analyses have shown that among cancer patients, lacking a private vehicle decreases treatment odds [8]. Additionally, individuals with public insurance plans and individuals with no insurance were more likely to experience transportation delays in our study. This may be attributable to the increased costs of travel experienced by those with public or no insurance. In a study observing travel distance among those in need of HIV medical care, it was found that those with no insurance traveled further than those with public insurance [9]. In fact, a key policy recommendation for reducing transportation barriers in accessing healthcare includes insurance coverage that is inclusive of transportation [10].

### Strengths and Limitations

Strengths of this study are its usage of a nationally representative sample and a large sample size. Limitations include biases with self-reported data, additional confounders not included in this model, and the cross-sectional nature of the data, which prohibits casual inferences.

## Supplementary material

10.2196/91207 Multimedia Appendix 1 Definition of knee arthritis and transportation delays.

## Abbreviations

NHIS: National Health Interview Survey
GED: General Education Development
aOR: Adjusted Odds Ratio
