# Supplementary material for: Transportation as a Barrier to Health Care Access Among Patients With Knee Arthritis: Cross-Sectional Study
Source: JMIR Form Res. 2026 Mar 6;10:e91207. doi: 10.2196/91207 (PMC12978929; doi:10.2196/91207)
Supplement: Multimedia Appendix 1 [file formative-v10-e91207-s001.docx]

Appendix I

*Arthritis was defined by the following question among respondents who were told “by a doctor or other health professional that [they had] some form of arthritis, rheumatoid arthritis, gout, lupus, or fibromyalgia.”: “During the past 30 days, have you had symptoms of pain, aching, or stiffness in or around a joint.” A second question prompted individuals to identify the knee as the affected joint.

†Transportation delays were defined by adults who agreed to the statement: “There are many reasons people delay getting medical care. Have you delayed getting care for [fill: alias] for any of the following reasons IN THE PAST 12 MONTHS…You didn’t have transportation.”
